# Use of Social Media Across US Hospitals: Descriptive Analysis of Adoption and Utilization

**DOI:** 10.2196/jmir.3758

**Published:** 2014-11-27

**Authors:** Heather M Griffis, Austin S Kilaru, Rachel M Werner, David A Asch, John C Hershey, Shawndra Hill, Yoonhee P Ha, Allison Sellers, Kevin Mahoney, Raina M Merchant

**Affiliations:** ^1^Penn Social Media and Health Innovation LabUniversity of PennsylvaniaPhiladelphia, PAUnited States; ^2^Department of Emergency MedicineHighland HospitalOakland, CAUnited States; ^3^Center for Health Equity Research and Promotion, Philadelphia VA Medical CenterDivision of General Internal Medicine, Perelman School of MedicineUniversity of PennsylvaniaPhiladelphia, PAUnited States; ^4^Penn Medicine Center for Healthcare InnovationPhiladelphia VA Medical CenterUniversity of PennsylvaniaPhiladelphia, PAUnited States; ^5^Penn Social Media and Health Innovation LabThe Wharton SchoolUniversity of PennsylvaniaPhiladelphia, PAUnited States; ^6^Penn Social Media and Health Innovation LabPerelman School of MedicineUniversity of PennsylvaniaPhiladelphia, PAUnited States; ^7^Penn MedicineUniversity of PennsylvaniaPhiladelphia, PAUnited States; ^8^Penn Social Media and Health Innovation LabDepartment of Emergency MedicineUniversity of PennsylvaniaPhiladelphia, PAUnited States

**Keywords:** social media, Internet, health information

## Abstract

**Background:**

Use of social media has become widespread across the United States. Although businesses have invested in social media to engage consumers and promote products, less is known about the extent to which hospitals are using social media to interact with patients and promote health.

**Objective:**

The aim was to investigate the relationship between hospital social media extent of adoption and utilization relative to hospital characteristics.

**Methods:**

We conducted a cross-sectional review of hospital-related activity on 4 social media platforms: Facebook, Twitter, Yelp, and Foursquare. All US hospitals were included that reported complete data for the Centers for Medicare and Medicaid Services Hospital Consumer Assessment of Healthcare Providers and Systems survey and the American Hospital Association Annual Survey. We reviewed hospital social media webpages to determine the extent of adoption relative to hospital characteristics, including geographic region, urban designation, bed size, ownership type, and teaching status. Social media utilization was estimated from user activity specific to each social media platform, including number of Facebook likes, Twitter followers, Foursquare check-ins, and Yelp reviews.

**Results:**

Adoption of social media varied across hospitals with 94.41% (3351/3371) having a Facebook page and 50.82% (1713/3371) having a Twitter account. A majority of hospitals had a Yelp page (99.14%, 3342/3371) and almost all hospitals had check-ins on Foursquare (99.41%, 3351/3371). Large, urban, private nonprofit, and teaching hospitals were more likely to have higher utilization of these accounts.

**Conclusions:**

Although most hospitals adopted at least one social media platform, utilization of social media varied according to several hospital characteristics. This preliminary investigation of social media adoption and utilization among US hospitals provides the framework for future studies investigating the effect of social media on patient outcomes, including links between social media use and the quality of hospital care and services.

## Introduction

Nearly three-quarters of adult Internet users in the United States use social networking sites [1]. Businesses have invested considerable resources in engaging consumers through these online platforms to enhance their reputation, brand recognition, and consumer loyalty. Similar strategies may be taken by hospitals, yet little is known about the extent to which hospitals use social media platforms [2-4].

Hospitals may adopt social media strategies to improve market share, profitability, or to advance their missions in health and health care [5-7]. A strong social media presence may support hospitals’ reputations and ability to attract patients. For example, patients may perceive hospitals with social media activity to be more likely to offer advanced technologies and cutting-edge therapies.

However, hospitals may not have control over the conversation on social media that surrounds their Web presence [8]. Much of the content on social media is generated by hospitals’ communities, including patients and their families, neighbors, employees, and potentially even competitors [9]. For example, social media sites such as Facebook and Yelp have empowered patients and their families to publicly rate their health care experience [10-17]. Although such ratings lack the systematic collection and analysis of data possible with carefully designed surveys, they happen organically, create no additional cost, and may provide some valuable signals about the markets or missions of health care organizations [7,18]. Indeed, Facebook “likes” in 1 urban region were associated with patients’ recommending a particular hospital and negatively associated with 30-day mortality rates [17]. Another study demonstrated that consumer ratings for hospitals on the social media website Yelp were associated with the more traditional hospital performance measures of patient experience of care generated by the Hospital Consumer Assessment of Healthcare Providers and Systems (HCAHPS) survey [10].

The relationships between hospital-associated social media activity, patient choices, clinical processes and outcomes, and hospital profit margins are unknown and almost certainly evolving rapidly. At the same time, it has become increasingly critical to find effective ways of communicating with patients outside of clinical settings. Mail and telephone communication channels that dominated the past are being supplemented or replaced by new media channels, and this is occurring faster in some demographic segments and hospitals than others [4,19]. In this study, we sought to describe the adoption and utilization of social media among US hospitals and determine whether adoption and utilization varied by hospital characteristics. This lays the groundwork for relating hospital social media adoption and utilization to other outcomes, including health care quality, market share, and profitability.

## Methods

### Study Design

We conducted a cross-sectional review of hospital-related activity on 4 of the most popular social media platforms: Facebook, Twitter, Foursquare, and Yelp. For each platform, we reviewed the adoption and utilization of social media among US hospitals.

### Study Population

We included all US hospitals reporting complete data to both the Centers for Medicare and Medicaid Services (CMS) HCAHPS survey and the 2010 American Hospital Association Survey (AHAS) [20,21]. The study cohort included 3371 US hospitals. We excluded hospitals operated by the federal government and those not classified as general medical and surgical centers, such as pediatric hospitals, psychiatric hospitals, specialty surgery centers, and long-term acute care hospitals. Because these hospitals provide care for specific subpopulations of patients, social media adoption and utilization may reflect specific types of care from different types of patients than the general population receiving care from hospitals that provide a wide range of services. Hospital characteristics were derived from the AHAS, including ownership/profit status (public, private nonprofit, private for-profit), teaching status (yes/no), urban designation (yes/no), bed count (small: less than 99 beds; medium: 100 to 299 beds; large: 300 or more beds), and region (northeast, midwest, west, south).

We extracted data for each hospital from the 4 social media platforms. Data included whether each hospital had an account (adoption) and, if so, activity on each social media account (utilization). These platforms were selected because of their widespread popularity, free public access, and availability of posted usage metrics.

Webpages on Facebook and Twitter are created by hospitals. Hospitals can create accounts and then post messages and pictures through these accounts to their followers. Facebook is a social networking platform that allows individuals and organizations to post and discuss content [22]. This content can be “liked” by users and shared with others. Facebook has 1.19 billion monthly active users worldwide [23]. Twitter is a microblogging site that allows individuals and organizations to post 140-character messages, or “tweets” [24]. Twitter has more than 230 million monthly active users who collectively generate 500 million tweets each day [25].

Webpages on Foursquare and Yelp, however, are not created by hospitals. Social media users create and generate the content of webpages for hospitals on these platforms. Foursquare is a location-based service application that allows individuals to “check-in” and indicate their presence at a geographic location [26]. Foursquare has more than 45 million users and more than 5 billion posted check-ins [27]. Yelp is an online rating platform where individuals can post reviews and comments about businesses [28]. Yelp has more than 100 million monthly unique users and over 47 million local reviews [29].

### Data Collection

To extract data from the 4 social media platforms, we first identified the home page for each hospital through an Internet search engine using hospital names from the HCAHPS and AHAS surveys. We then followed posted links to social media webpages. If the hospital website did not feature links to social media webpages, direct searches using the hospital name were performed on the search function provided by the social media platform. In these cases, the identity of each hospital’s social media webpage was confirmed by matching the address of the hospital on the social media page with the known address of the hospital from the HCAHPS and AHAS surveys.

We defined *adoption* to be whether or not a hospital had a social media account. We defined *utilization* to be metrics of social media user activity or content that could be extracted from each social media webpage. These included number of likes (Facebook), number of followers (Twitter), number of check-ins (Foursquare), and number of reviews (Yelp).

For social media webpages attributed to multiple hospitals in a consortium or network, adoption and utilization of the network social media page was attributed to each network hospital. For hospitals with multiple social media pages on 1 platform, we selected either the page endorsed by the hospital or the page with the greatest volume of social media activity. Social media webpages were reviewed over a 1-month period (August 2014).

### Statistical Analysis

We report the percentage of hospitals having Facebook, Twitter, Foursquare, and Yelp to show the adoption of social media platforms across hospitals. Because of the right-skewed distribution of utilization (likes, followers, check-ins, and reviews), we report medians and IQRs. We used the Mood median test to determine differences in the magnitude of social media utilization between groups of hospitals with different characteristics. We used ordinary least squares (OLS) regressions to assess the independent associations of hospital characteristics on the magnitude of social media activity. Due to the skewed nature of utilization, we used the log transformation of social media utilization to approximate the normal distribution. The variance inflation factor and normality of residuals indicated OLS regression was appropriate for these outcomes. For all analyses, a *P* value <.05 was considered statistically significant. We performed sensitivity analyses to assess the effect of attributing 1 hospital’s social media page adoption and utilization characteristics to all hospitals in a network and all associations presented were unchanged. Therefore, data are presented such that each webpage represents a unique hospital. All statistical analyses were performed using STATA version 10.0 (StataCorp, College Station, TX, USA). To display the geographic distribution of social media utilization across hospitals, we geocoded each hospital based on street address in ArcGIS version 10.1 (ESRI, Redlands, CA, USA).

## Results

### Adoption

Of the total 3371 US hospitals identified, the adoption of social media websites varied across platforms, with 3351 (99.41%) having a Facebook, 1713 (50.82%) having a Twitter, 3351 (99.41%) having a Foursquare, and 3342 (99.14%) having a Yelp account. Overall, 1699 (50.40%) hospitals had accounts on all 4 platforms. Few hospitals (42/3371, 1.25%) used just 1 or 2 types of social media platform.

### Utilization

**Figure 1:**
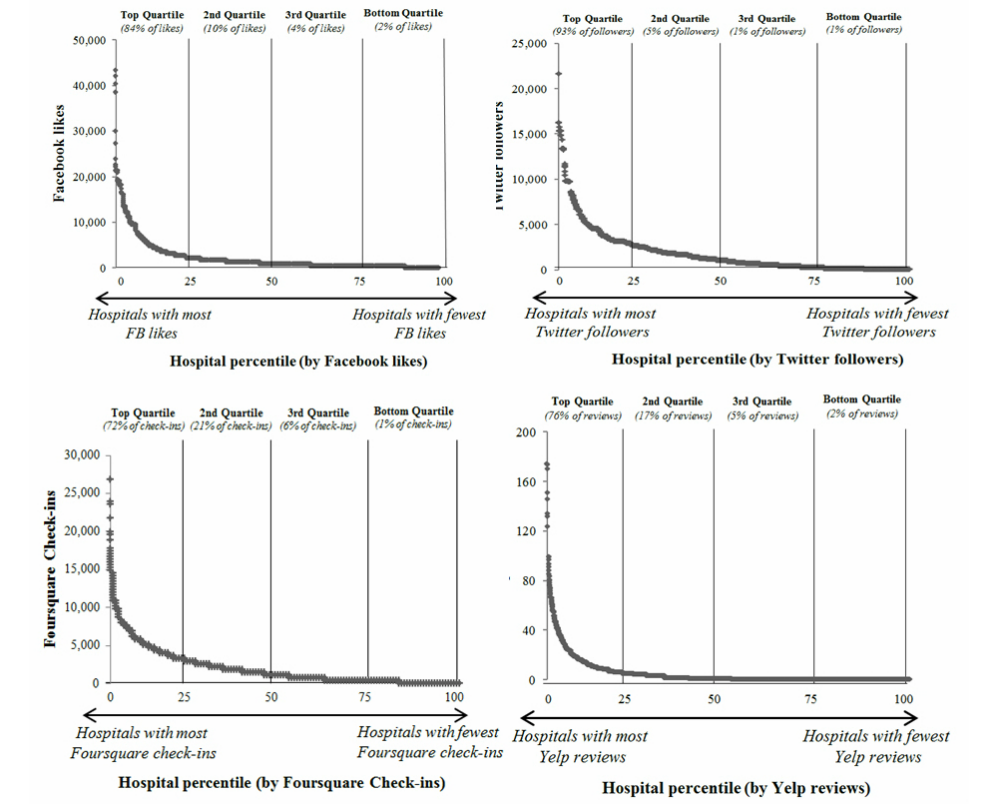
Distribution of utilization of social media across US hospitals.

The distribution of social media utilization for US hospitals was right-skewed for all social media platforms (Figure 1). Hospitals in the top quartile accounted for more than 68% of likes, followers, check-ins, and reviews. This figure shows the relationship between utilization (likes, followers, check-ins, and reviews) on the y-axis and hospital percentile on the x-axis.

**Figure 2:**
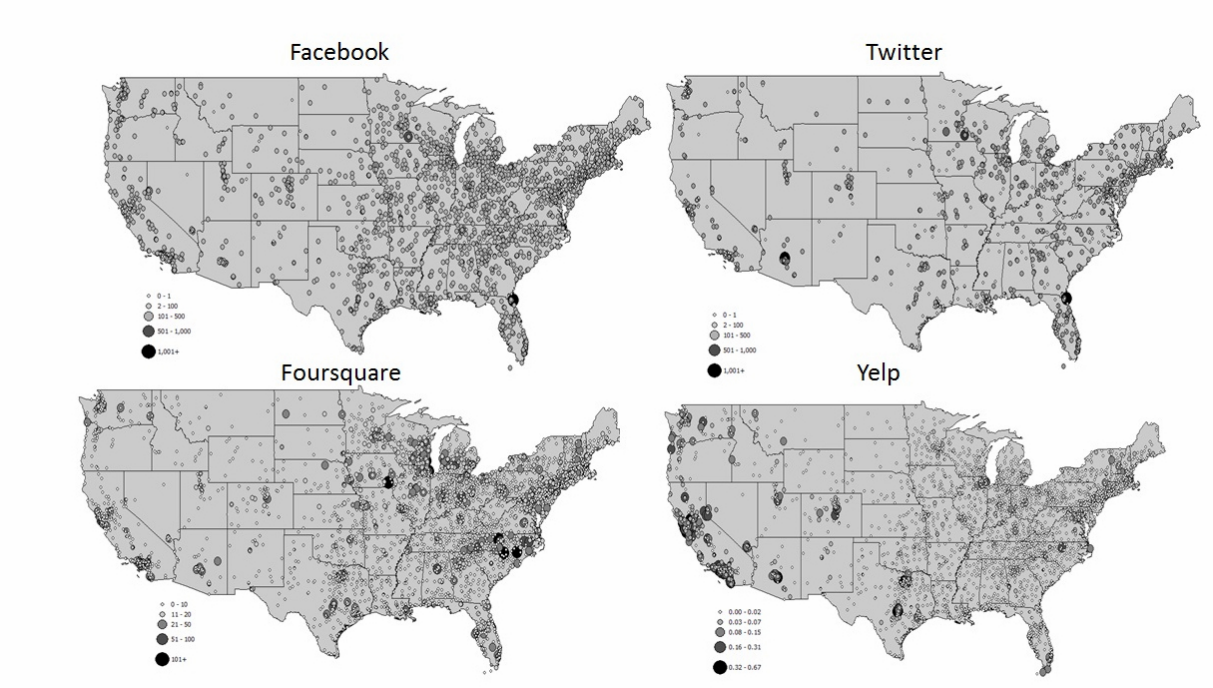
Maps of social media utilization for hospitals adjusted by bed count.

The geographic distribution of social media utilization adjusted for the size of the hospital (using bed count) also varied (Figure 2). All social media platforms appeared widely spread across the United States with a higher density of hospitals using social media in urban areas. The northeast United States had a large cluster of hospitals using social media, but the west also had clusters of hospitals with high Foursquare and Yelp utilization. This figure illustrates the number of likes, followers, check-ins, and reviews by hospital (each dot represents a hospital location).

To better understand hospital characteristics associated with this variation, Tables 1 and 2 display the magnitude of social media utilization differentiated by hospital characteristics.

Larger, urban, private nonprofit, and teaching hospitals had significantly more social media utilization than their comparison groups across all 4 social media platforms. For example, large hospitals (>300 beds) compared to the smallest hospitals (<99 beds) had a median 2817.5 (IQR 1289-5533) versus median 425.5 (IQR 133-1127) Facebook likes, median 1409 (IQR 525-3115) versus median 753 (IQR 164-2381) Twitter followers, median 4595 (IQR 2383-7321) versus median 212 (IQR 79-539) Foursquare check-ins, and median 5 (IQR 1-17) versus median 0 (IQR 0-1) reviews on Yelp. Urban hospitals had a median 1409 (IQR 509-3453) versus median 518 (IQR 151-1199.5) Facebook likes for rural hospitals, median 1130 (IQR 392.5-2860.5) versus median 491 (IQR 118-1771) Twitter followers, median 2027.5 (IQR 765.5-4180.5) versus median 211 (IQR 79-537) Foursquare check-ins, and median 2 (IQR 0-9) versus median 0 (IQR 0-0) Yelp reviews.

**Table 1:** Magnitude of social media utilization relative to hospital characteristics: Facebook and Twitter.^a^

Social media platform		Facebook likes (n=3351)		Twitter followers (n=1713)	
		Median	IQR	Median	IQR
**Region**					
	Northeast	1248	451-3015	825	251-2748
	Midwest	931	332-2780	1200.5	367.5-2818
	West	813	261-1971	882	283-2673
	South	1060	276-2767	854	268-2411
**Urban**					
	Yes	1409	509-3453	1130	392.5-2860.5
	No	518	151-1199.5	491	118-1771
**Bed count**					
	Small (<99)	425.5	133-1127	753	164-2381
	Medium(100-299)	1062	418.5-2196.5	779	250-2390
	Large(300+)	2817.5	1289-5533	1409	525-3115
**Profit status**					
	Public	712	227-2023	650	136-220
	Private nonprofit	1302	473-3240	1202	409-3104
	Private for-profit	426.5	151-1302	415	120-1019
**Teaching hospital**					
	Yes	4155	1854-8715	2706	1078-5093
	No	900	281-2203	817	261-2381

^a^ Within each characteristic, Mood median tests indicate at least one median is significantly different at the alpha .05 level.

**Table 2:** Magnitude of social media utilization relative to hospital characteristics: Foursquare and Yelp.^a^

Social media platform		Foursquare check-ins (n=3351)		Yelp Reviews (n=3342)	
		Median	IQR	Median	IQR
**Region**					
	Northeast	2324	847-4865	2	0-7
	Midwest	851	223-2941	0	0-2
	West	1083	271-2655	1	8-27.5
	South	673.5	155.5-2124.5	0	0-3
**Urban**					
	Yes	2027.5	765.5-4180.5	2	0-9
	No	211	79-537	0	0-0
**Bed count**					
	Small (<99)	212	79-539	0	0-1
	Medium (100-299)	1437	560-2633	1	0-6
	Large (300+)	4595	2383-7321	5	1-17
**Profit status**					
	Public	375	109.5-1623	0	0-2
	Private nonprofit	1465	349-3550	1	0-6
	Private for-profit	732	185-1733		
**Teaching hospital**					
	Yes	2698	1032-4913	4	1-15
	No	809	245-2323	0	0-3

^a^ Within each characteristic, Mood median tests indicate at least one median is significantly different at the alpha .05 level.

Tables 3 and 4 report the results of OLS regressions to assess the independent associations between hospital characteristics and the magnitude of social media utilization. Each regression model explained significantly more variance in the outcome than was left unexplained (Facebook: *F*_9,3068_=106.44, *P*<.001; Twitter: *F*_9,1549_=32.84, *P*<.001; Foursquare: *F*_9,3334_, *P*<.001; Yelp: *F*_9,3330_=293.43, *P*<.001). Hospital characteristics explained 23.8% of the variation in Facebook, 16.0% of Twitter, 53.82% of Foursquare, and 38.95% of Yelp utilization. Urban and teaching hospitals tended to have more social media utilization. Different regions displayed different utilization of the 4 social media platforms. The magnitude of social media activity increased with hospital size, significantly for Yelp and Facebook. Private for-profit hospitals had significantly fewer Facebook likes compared to public (*P*<.001) or private nonprofit hospitals compared to public hospitals (*P*<.001), and this association was the same for the number of Twitter followers.

**Table 3:** Ordinary least squares regression of social media utilization relative to hospital characteristics: Facebook and Twitter.

Social media platform		Facebook likes (n=3351)			Twitter followers (n=1713)		
		Coefficient	SE	*P*	Coefficient	SE	*P*
**Region**							
	Northeast (ref)						
	Midwest	0.388	0.084	<.001	0.470	0.107	<.001
	West	0.003	0.092	.97	0.397	0.117	<.001
	South	0.467	0.085	<.001	0.521	0.110	<.001
**Urban**							
	Yes	0.546	0.066	<.001	0.693	0.100	<.001
	No (ref)						
**Bed count**							
	Small (ref)						
	Medium	0.639	0.066	<.001	–0.014	0.095	.88
	Large	1.176	0.088	<.001	0.123	0.114	.28
**Profit status**							
	Public (ref)						
	Private nonprofit	0.494	0.077	<.001	0.759	0.117	<.001
	Private for-profit	–0.465	0.094	<.001	–0.430	0.146	.003
**Teaching hospital**							
	Yes (ref)						
	No	–0.792	0.112	<.001	–0.835	0.128	<.001
Constant		6.091	0.143	<.001	5.945	0.187	<.001
*R* ^*2*^		0.238			0.160		

**Table 4:** Ordinary least squares regression of social media utilization relative to hospital characteristics: Foursquare and Yelp.

Social media platform		Foursquare check-ins (n=3351)			Yelp Reviews (n=3342)		
		Coefficient	SE	*P*	Coefficient	SE	*P*
**Region**							
	Northeast (ref)						
	Midwest	–0.118	0.065	.07	–0.254	0.077	<.001
	West	–0.463	0.071	<.001	1.190	0.074	<.001
	South	–0.566	0.065	<.001	–0.084	0.076	.27
**Urban**							
	Yes	1.179	0.051	<.001	0.910	0.079	<.001
	No (ref)						
**Bed count**							
	Small (ref)						
	Medium	1.251	0.051	<.001	0.495	0.071	<.001
	Large	2.015	0.069	<.001	0.829	0.083	<.001
**Profit status**							
	Public (ref)						
	Private nonprofit	0.361	0.060	<.001	0.269	0.078	<.001
	Private for-profit	0.082	0.073	.26	0.121	0.093	.19
**Teaching hospital**							
	Yes (ref)						
	No	–0.564	0.088	<.001	–0.544	0.085	<.001
Constant		5.461	0.111	<.001	0.374	0.128	.004
*R* ^*2*^		0.538				0.390	

## Discussion

### Principal Findings

In this study, we examined the extent to which US hospitals had social media platforms and then determined utilization of each social media platform by systematically extracting data from hospital social media webpages This paper has 3 central findings: (1) adoption of social media is widespread among US hospitals, (2) hospitals are adopting different social media platforms, and (3) social media utilization is variable with larger, urban, private nonprofit, and teaching hospitals tending to demonstrate more activity. Laying the exploratory foundation for future research regarding hospital social media use, this study can inform the potential link between social media use and hospital quality.

### Adoption of Social Media by Hospitals Is Widespread

Compared to the results of prior studies, our results demonstrate a dramatic growth of social media adoption among hospitals. In a random sample of US hospitals, 21% of hospitals used social media in 2010 [4]. At the time, 18% of hospitals maintained a Facebook account and 16% had a Twitter account [4]. Three years later, our study demonstrates significantly higher percentages of hospitals with social media accounts—more than 90% have Facebook, Foursquare, and Yelp accounts, and approximately 40% have a Twitter account. In particular, a significantly higher proportion of hospitals in rural locations (93.9%) and smaller hospitals (94.4%) have a Facebook account compared to the 2010 report [4]. Additionally, a study of hospitals in Western Europe showed that social media use is growing, with Facebook being the most popular social media platform—67.0% of hospitals in Western Europe had a Facebook account [30]. This dramatic increase in social media use may show the increasing value of social media to hospitals to potentially improve market share, engage with patients, increase profitability, or advance their missions in health and health care [5-7].

### Hospitals’ Adoption of Social Media Varies Across Social Media Platforms

Our study also demonstrates that adoption varied by social media platform, with more hospital-generated accounts on Facebook than Twitter, and more public-generated accounts for Foursquare and Yelp. As adoption in this context reflects whether or not a hospital set up an account, utilization (measured by likes and followers) allows for a better understanding of how actively hospitals are using their accounts and how actively the public is responding to their content.

Although it is unknown which platform may best connect hospitals with patients and for what purpose, it is probable that users will continue to interact with hospitals through social media, even with the continual introduction of new social media portals, such as Instagram, Pinterest, and Snapchat. Particularly for Facebook and Twitter, these accounts may enable hospitals to engage in dialog with patients, share knowledge, and solicit patient opinions [2,11].

Yelp presents an interesting platform for hospitals to gauge patient and public experiences and opinions, which may be helpful when thinking about hospital quality and patient perception [10]. Because Yelp reviews can be collected in real time, hospitals can collect reviews and relate them to quality and quality surveys, including the HCAHPS. Yelp reviews are related to traditional hospital performance measures [10]; therefore, reviews may also be helpful to find measures that are more important to patients. Also, reviews may highlight potential areas that hospitals are not surveyed about but are still important to patients, such as how family members perceive quality of care.

Facebook is also an interesting social media platform that hospitals may use to increase reputation and attract patients. With the ability of hospitals to respond to comments made by Facebook users on the hospital’s website, dialogs between hospitals and patients could foster important conversations regarding quality of care that traditional surveys may not have the ability to do [17]. Also, the ability to respond to patients in real time and collect data in real time for not only Facebook but all social media platforms provides the ability for hospitals to potentially assess quality and other metrics faster than traditional survey formats.

### Social Media Use Varies Widely Across Hospital Characteristics

Additionally, the utilization of social media among hospitals varies across hospital characteristics. Large, urban, private nonprofit, and teaching hospitals tend to have more likes, followers, check-ins, and reviews. These hospitals may have more hospital communications personnel dedicated to social media presence and engagement, different policies regarding social media use by the hospital, or more resources dedicated to outreach and communication via social media. As a way to increase social media presence and extend reach in social media, hospitals could be more active, such as increase tweeting on Twitter or posting to Facebook. Specific to Twitter, the number of followers is significantly correlated with the number of tweets (ρ=.113, *P*<.001), so more activity could lead to more followers, resulting in greater social media presence. Future research could investigate why some hospitals tend to post more than others do and social media use as a patient engagement tool.

Additionally, certain hospitals are outliers with comparatively higher social media activity. For example, several hospitals had more than 400,000 Facebook likes and Twitter followers. A potential explanation for high numbers of likes and followers may be the popularity and frequency of content disseminated on these pages, which the public deems valuable enough to share with their own social networks. Social media engagement may provide a measure of the value of information services that hospitals offer to patients, providers, policymakers, and their online community. A better understanding of the benefits of social media engagement and the approaches used by outliers to increase visibility could be useful for hospitals at the early stages of creating social media accounts.

### Limitations

This study has several limitations. Our findings represent a snapshot of hospital adoption and utilization. Social media are changing rapidly and so are the media channels themselves. The same speed with which these channels are adopted and new channels are developed reveals the importance of examining how they are used by hospitals. In addition, some hospitals in networks share social media accounts. In this case, we attributed the social media account adoption and utilization to all hospitals within the network. However, after conducting sensitivity analyses that included and excluded all the hospitals in networks, the results remained the same. Lastly, there may be hospital social media webpages that we did not locate using our search methods. This may lead to underestimation of social media adoption. Our search method, however, mimics the strategy that the public might use to search for the social media webpage for a particular hospital.

### Conclusion

Adoption of certain social media platforms is widespread among US hospitals, is greater than in previous reports, and remains varied. The functional purpose of social media use by hospitals and its opportunity and impact on patients and populations remains largely unknown. Nevertheless, the tremendous reach of these new media and their ability to harness existing networks with established trust relationships suggests they have the potential to become dominant communication channels for health care.

## Abbreviations

AHAS: American Hospital Association Survey
HCAHPS: Hospital Consumer Assessment of Healthcare Providers and Systems
OLS: ordinary least squares
